# Measurement Method of Magnetic Field for the Wire Suspended Micro-Pendulum Accelerometer

**DOI:** 10.3390/s150408527

**Published:** 2015-04-13

**Authors:** Yongle Lu, Leilei Li, Ning Hu, Yingjun Pan, Chunhua Ren

**Affiliations:** 1 Key Lab. of Opto-Electronic Technology & System, Ministry of Education, Chongqing University, Chongqing 400044, China; E-Mails: luyongle08@163.com (Y.L.); pyj@cqu.edn.cn (Y.P.); rchht@cqu.edu.cn (C.R.); 2 State Key Lab. of Information Engineering in Surveying, Mapping and Remote Sensing, Wuhan University, Wuhan 430079, China; 3 College of Aerospace Engineering, Chongqing University, Chongqing 400044, China; E-Mail: ninghu@cqu.edu.cn

**Keywords:** Wire Suspended Micro-Pendulum Accelerometer, magnetic field strength, permanent magnet, iterative least squares

## Abstract

Force producer is one of the core components of a Wire Suspended Micro-Pendulum Accelerometer; and the stability of permanent magnet in the force producer determines the consistency of the acceleration sensor’s scale factor. For an assembled accelerometer; direct measurement of magnetic field strength is not a feasible option; as the magnetometer probe cannot be laid inside the micro-space of the sensor. This paper proposed an indirect measurement method of the remnant magnetization of Micro-Pendulum Accelerometer. The measurement is based on the working principle of the accelerometer; using the current output at several different scenarios to resolve the remnant magnetization of the permanent magnet. Iterative Least Squares algorithm was used for the adjustment of the data due to nonlinearity of this problem. The calculated remnant magnetization was 1.035 T. Compared to the true value; the error was less than 0.001 T. The proposed method provides an effective theoretical guidance for measuring the magnetic field of the Wire Suspended Micro-Pendulum Accelerometer; correcting the scale factor and temperature influence coefficients; *etc.*

## 1. Introduction

Pendulum accelerometers are widely used in the measurement of acceleration, force, vibration and shock [1,2,3,4,5,6,7]. There are two different types of pendulum accelerometers whose basic principle is either an external inertial force and the responded elastic force on a proof mass are rebalanced at a new position [8,9], or a feedback force keeps the proof mass at its original position [10,11]. Accordingly, an acceleration value could be measured by the deformation or the feedback, representing by a current [8,9,10,11]. The most used methods to generate a feedback force are electrostatic and electromagnetic induction. The Electrostatic Balanced Accelerometer has a super high accuracy and thus is widely used in micro-gravity space missions, like the measurement of Earth’s gravity field [11,12,13]. The Electromagnetic Balanced Accelerometer, though not comparable to the electrostatic accelerometer, improves a lot in the accuracy and measurement range as the processing technology develops. One example is the Quartzose Flexible Accelerometer (QFA), which has a high accuracy and anti-interference ability in low-frequency and low-g environments [14]. On the other hand, the miniaturization of electromagnetic accelerometer allows it to possess smaller sizes and lower costs [10]. The Wire Suspended Pendulum Accelerometer is such a micro-sensor. Its suspension is a very thin wire and other components also have a compact designed.

The conventional accelerometer is composed of a spring, a damper and a proof mass, which in fact is a second-order single degree of freedom vibration system [13,15]. In a different structure, the Wire Suspended Micro-Pendulum Accelerometer works at a closed-loop feedback mode, theoretically leading to higher sensitivity and stability [10,16,17]. However, the errors of this accelerometer, such as the nonlinear scale factor and temperature coefficient, restrict its wide applications [13]. Amin *et al.* [18], Ren *et al.* [19], and Yasir *et al.* [20] compensated the global error of the accelerometer from systemic prospect, while Li fitted and then removed the scale factor and bias separately [21]. All above models are software compensation without knowing the error mechanism.

An accelerometer needs to be calibrated before use since its scale factor and temperature coefficient change slowly with time. The Wire Suspended Micro-Pendulum Accelerometer works at a homeostasis state, *i.e.*, the balance of inertial moment and feedback moment inside the sensor. For an assembled accelerometer, the dimension and mass of the proof do not change, therefore the scale factor and temperature coefficient are primarily related to the feedback moment. Permanent magnet is the core component to induce the feedback moment, and determines the stability of scale factor and temperature coefficient in a great extent. Since the demagnetization rate of a permanent magnet may vary one by one due to strong individual difference [22], accurate measurement of magnetic field strength is crucial for the calibration of the sensor. However, it is in fact a very intractable problem when facing a micro-sensor. The reason is, in order to generate a strong and uniform magnetic field, the space between two magnets is designed very limited just to accommodate the coil. Naturally, it is too small for the magnetometer probe to conduct direct measurements.

In this work, we investigated a new indirect method to measure the magnetic field strength of a Wire Suspended Micro-Pendulum Accelerometer. The method is based on the working principle of the accelerometer, using the sensor output at several different scenarios, including known accelerations and temperatures as combinations, to identify the remnant magnetization of magnet. Iterative Least Squares algorithm was used for the adjustment of the data due to the inherent nonlinearity in this measurement problem. The calculated remnant magnetization was 1.035 T. Compared to the true value, the error was less than 0.001 T. The present results verify the effectiveness of the proposed identification model and testing procedures.

## 2. Wire Suspended Micro-Pendulum Accelerometer

**Figure 1:**
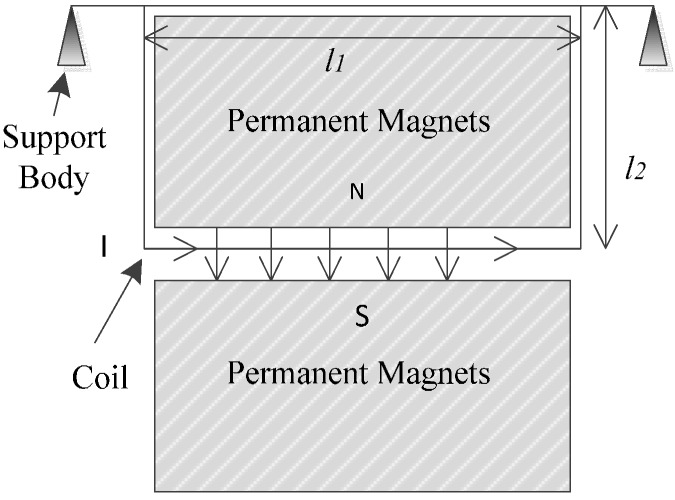
Schematic view of the Wire Suspended Micro-Pendulum Accelerometer.

Figure 1 shows the schematic diagram of the Wire Suspended Micro-Pendulum Accelerometer. A pair of permanent magnets builds a stable magnetic field, in which the sensing side of a rectangular coil is suspended to cut magnetic line of force, and the other long side is supported by the body. When there is acceleration orthogonal to the coil plane, the sensing side will deviate from its original position with respect to the supporting side. This deviation is detected and transferred to current by a displacement transducer. As a consequence, the current-carrying coil in magnetic field experiences an Ampere Force, which pushes the coil back to its original position. It is noticed that the rebalance is a transient process and the sensing side of the coil swings in a very little amplitude. By measuring the current in the coil, we can easily have the numeric information of the acceleration.

**Figure 2:**
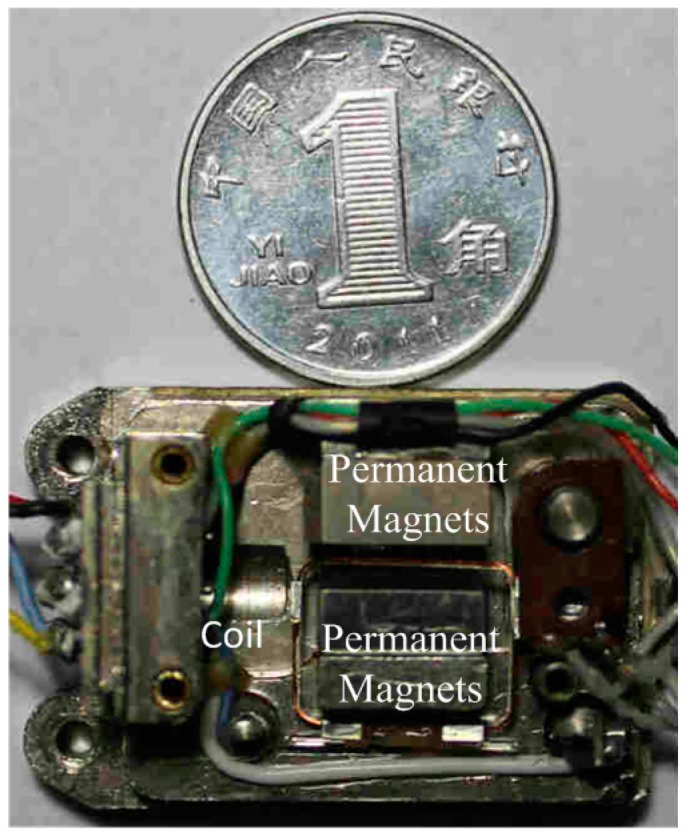
A Wire Suspended Micro-Pendulum Accelerometer.

The trial Wire Suspended Micro-Pendulum Accelerometer is shown in Figure 2. A pair of SmCo permanent magnets was mounted on the base of the sensor in parallel. SmCo (SmCoCYRS24) has some good properties as a magnetic source, like high magnetic energy product, intrinsic coercivity and relatively good temperature stability. In order to generate a strong and uniform magnetic field, without increasing the magnet dimension, the best scheme is to reduce the space between the two magnets. In the trial accelerometer, the distance between two magnets was 1 mm, *i.e.*, a compromised value to have wanted magnetic field and being spacious for coil swinging.

When there is an acceleration input in the sensing direction, the inertial moment on the coil is:

(1) $$\tau_{1}=ml_{1}al_{2}+2\int_{0}^{l_{2}}\max dx=ml_{1}al_{2}+mal_{2}^{2}$$

where τ_1_ represents the inertial moment, *a* is the acceleration in the sensing direction, *m* is the surface density of the coil, and *l*_1_ and *l*_2_ correspond to the lengths of the coil’s sensing side and cantilever side, respectively (see Figure 1 for detailed dimension definition).

**Figure 3:**
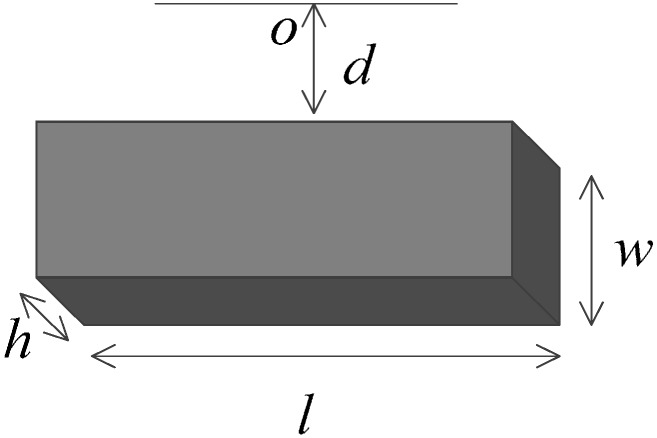
Dimension of the permanent magnet.

On the other hand, the moment on the coil caused by the Ampere Force is:

(2) $$\tau_{2}=2NBIl_{1}l_{2}$$

where τ_2_ represents the Ampere moment, *N* is the turn number of the coil, *B* is the strength of a signal permanent magnet and *I* is the current intensity. For the magnet with the dimension shown in Figure 3, the magnetic field strength of a single magnet could be derived according to Coulomb’s Law [23]:

(3) $$B=\frac{B_{r}}{\pi }\left(\arctan\frac{lh}{2d\sqrt{l^{2}+h^{2}+4d^{2}}}-\arctan\frac{lh}{2(d+w)\sqrt{l^{2}+h^{2}+4{\left(d+w\right)}^{2}}}\right)$$

where *B_r_* represents the remnant magnetization, *l*, *h* and *w* correspond to the length, height and width of the permanent magnet, respectively, and *d* is the distance from magnet to a point *O* in space. The magnetic field follows the vector superposition principle, thus the working strength in the sensor is the vector sum of two magnets.

The temperature coefficient cannot be ignored for most magnets with high magnetic energy product [24]. Reference [25] provides the variation of SmCo remnant magnetization with temperature:

(4) $$B_{r}=B_{0}+\beta B_{0}(T-T_{0})$$

where β represents the temperature coefficient, *B_r_* is the working remnant magnetization, *B_0_* is the remnant magnetization at 24 °C, *T* is the working temperature and *T_0_* denotes the normal temperature, 24 °C. Obviously, there exists a linear relationship between remnant magnetization and temperature.

By substituting Equation (4) into Equation (3), we can have the strength formula of magnetic field in full temperature range:

(5) $$B=\frac{B_{0}+\beta B_{0}(T-T_{0})}{\pi }\left(\arctan\frac{lh}{2d\sqrt{l^{2}+h^{2}+4d^{2}}}-\arctan\frac{lh}{2(d+w)\sqrt{l^{2}+h^{2}+4{\left(d+w\right)}^{2}}}\right)$$

The coil is in a dynamic balanced state when working. When the external acceleration changes, the coil deviates from the balanced position. This deviation is transferred to current by a displacement transducer and therefore generates an Ampere moment under the effect of magnetic field. When the resultant of Ampere moment and inertial moment equals to zero, the coil goes back to the balanced state. By combining Equations (1) and (2), we have:

(6) $$ml_{1}al_{2}+mal_{2}^{2}+2NBIl_{1}l_{2}=0$$

Rewriting Equation (6) in the form of acceleration input and current output, plus considering the current bias and stochastic error, leads to:

(7) $$I=-\frac{M}{4NBl_{1}}\cdot a+I_{0}+v$$

where *M* = 2*ml*_1_ + 2*ml*_2_ represents the mass of coil, *I*_0_ is the current bias, and *v* is the measurement noise, which normally meets Gauss normal distribution *v* ~ (0, σ^2^). The current output is proportional to the acceleration, since all other parameters are given. By measuring the current, the acceleration is easily obtained by dividing a scale factor.

Substituting Equation (5) into Equation (7) leads to the input-output model in full temperature range:

(8) $$I=k\frac{a}{(1+\beta \Delta T)B_{0}}+I_{0}+v$$

where:

k=−M4Nl11π(arctanlh2dl2+h2+4d2−arctanlh2(d+w)l2+h2+4(d+w)2)

$$\Delta T=T-T_{0}$$

*k*/(1 + β*∆T*)*B*_0_ is the scale factor of the accelerometer. Temperature coefficient β of SmCo is a negative value, so the magnetic field strength will decrease if the working temperature increases, and as a result the scale factor increases.

## 3. Measurement Principle of Accelerometer’s Internal Magnetic Field

Remnant magnetization determines the magnetic field strength. However, it is not constant but changes slowly with time. Therefore, the scale factor changes. Normally we use demagnetization rate to quantify the changing rate of remnant magnetization. The problem is that the demagnetization rate is not accurate and may even shift in tough working environments, especially existence of high external magnetic fields and high shock. Moreover, the space between two magnets is so small that cannot use a magnetometer probe for direct measuring. Therefore, it is necessary to develop a new method to measure the magnetic field strength or the remnant magnetization.

Equation (8) explains that the acceleration could be resolved if we have known remnant magnetization, working temperature and output current. On the contrary, the remnant magnetization could be resolved as well, provided temperature, acceleration and current, were given. This is the basic principle for indirectly measuring the remnant magnetization.

The current has a nonlinear relationship with the remnant magnetization. In addition, it suffers from the bias and stochastic noise. To obtain a best resolution, we use Iterative Least Squares as the adjustment algorithm. By expanding Equation (8) into Taylor Series at the initial value of *B*_0_, and omitting second and higher order items, it can be rewritten in the form of error and observation:

(9) $$I-I_{0}-k\frac{a}{(1+\beta \Delta T)B_{0}^{\prime }}=-k\frac{a}{(1+\beta \Delta T){B_{0}^{\prime }}^{2}}\Delta B_{0}+v$$

where $B_{0}^{\prime }$ denotes the initial value or the estimation of *B*_0_, and Δ*B*_0_ is estimation error. Equation (9) gives the linear expression about estimation error of remnant magnetization and its observation. Suppose we made *n* times measurements, and the input-output of every measurement is (Δ*T_i_*, *a_i_*, *I_i_*), then Equation (9) can be expressed in the matrix form:

(10) y=Hx+V

where *y* represents the observation vector, *H* is Jacob matrix, *x* is the estimation error of remnant magnetization, and *V* is the observation error vector. Each term is given as follows:

(11) $$y=\left[I_{1}-I_{0}-\frac{ka_{1}}{(1+\beta \Delta T_{1})B_{0}^{\prime }}\;I_{2}-I_{0}-\frac{ka_{2}}{(1+\beta \Delta T_{2})B_{0}^{\prime }}\text{ L }I_{n}-I_{0}-\frac{ka_{n}}{(1+\beta \Delta T_{n})B_{0}^{\prime }}\;\right]$$

(12) $$H={\left[-k\frac{a_{1}}{(1+\beta \Delta T_{1}){B_{0}^{\prime }}^{2}}\;-k\frac{a_{2}}{(1+\beta \Delta T_{2}){B_{0}^{\prime }}^{2}}\text{ L }-k\frac{a_{n}}{(1+\beta \Delta T_{n}){B_{0}^{\prime }}^{2}}\;\right]}^{T}$$

(13) $$V={\left[v_{\text{1}}\;v_{\text{2}}\;\cdot \cdot \cdot \;v_{\text{n}}\;\right]}^{T}$$

The covariance matrix of the observation noise is:

(14) $$R=E(vv^{T})=\left[\begin{array}{l} \sigma_{1}^{2}\;L\;0\\ M\;M\\ 0\;L\;\sigma_{n}^{2} \end{array}\right]$$

Calculating the Least Squares resolution of Equation (10), and updating the estimation of remnant magnetization by correcting the error lead to:

(15) $$\hat{x}={\left(H^{T}R^{-1}H\right)}^{-1}H^{T}R^{-1}y$$

(16) $$B_{0}^{\prime }=B_{0}^{\prime }-x$$

If the initial value is not accurate enough, linearization error in Equation (9) is not negligible. By carrying out iterative calculation for Equation (9) to Equation (16) until convergence, we have the best estimation of remnant magnetization. The estimation error covariance is:

(17) $$Q={\left(H^{T}R^{-1}H\right)}^{-1}$$

As for the unknown current bias *I*_0_, we can linearize Equation (9) at the initial values of *B*_0_ and *I*_0_, and make estimation for both remnant magnetization and current bias. In this way, each measurement has the same weight; that is, the covariance matrix *R* has same diagonal values. However, current bias is usually a random constant; it varies at each time of powering on, and gets larger when temperature rising from our experience. In the measurement process, the sensor needs to be powered off for thermal exchanging when working temperature changes, and then powered on again after thermal balance. Consequently, there are several biases needing to be estimated. The *x* in Equation (10) becomes a multi-dimension vector (Δ*B*_0_ Δ*I*_1_ Δ*I*_2_ ···)*^T^* and the observability decreases. If the initial values are not accurate enough, the Iterative Least Squares algorithm is difficult to converge. Furthermore, noise *v*, in theory, is of more randomness when temperature is rising. The present model with equal weights is not coincident with the real situation.

From Equation (8), it is easy to understand that the output current equals the sum of bias and noise if the input acceleration is zero. When calculating the mean and variance of outputs at each temperature, the former is the bias in least squares sense, and the latter could be the elements in covariance matrix or the inverse of weights. By substituting the biases and variances into from Equation (9) to Equation (16), we can then perform iterative estimation for single variable *B*_0_. Meanwhile, the scale factor at any temperature can be calculated by *k*/(1 + β*∆T*)*B*_0_.

The calibration can also be carried out by conventional procedures without knowing the magnetic field strength, *i.e.*, the scale factor is calculated by dividing the given acceleration input from the current output according to Equation (8). It is simpler compared to indirect method proposed in the above paragraphs. However, remnant magnetization is the intrinsic reason why the sensor’s parameters are drifting. Examining the changing of the remnant magnetization helps us to have more knowledge about the magnet and improve our model. It is also reasonable to calculate the remnant magnetization in terms of data processing. The scale factor is a temperature-related parameter. It can only be calibrated at several discrete temperatures by conventional calibration method. Furthermore, the interpolation needs to be applied if the sensor is working at other temperatures. By using the method proposed in this paper, all the collected data, including different accelerations and temperatures inputs, can be built in one Least Squares equation, since the remnant magnetization is temperature irrelevant. It adjusts the measuring error in a global way and avoids the error introduced by the interpolation when calculate the scale factor. Moreover, it provides an evaluation criterion for the sensor’s condition. We can find out if the model (Equation (8)) still applies to the sensor by examining the residual of the Iterative Least Squares. If the Iterative Least Squares stopped converge at a large residual, then something wrong must happen to the sensor and it is not usable anymore.

## 4. Experiment

The measurement of remnant magnetization is based on the working principle of Wire Suspended Micro-Pendulum Accelerometer as shown in Equation (8). Given known acceleration and temperature inputs, the remnant magnetization could be identified by applying the Iterative Least Squares algorithm to the current output. Equation (17) illustrates that the algorithm accuracy depends on the covariance matrix *R* of noise and Jacobi matrix *H*. A simple way to improve the accuracy is to have a relatively large acceleration input when measuring noise, and other parameters in *H* are given (see Equation (12)). Thus, the accelerometer was placed in the vertical direction by a dividing head to have –*g* and +*g* acceleration input. Zero acceleration input was also tested, by aligning the sensor to the horizontal, to calculate the bias and covariance. For the testing temperature, we know from Equation (4) that the remnant magnetization is linearly correlated with it. In this sense, three typical temperature points, −40 °C, 24 °C, and 60 °C, were selected for testing.

**Figure 4:**
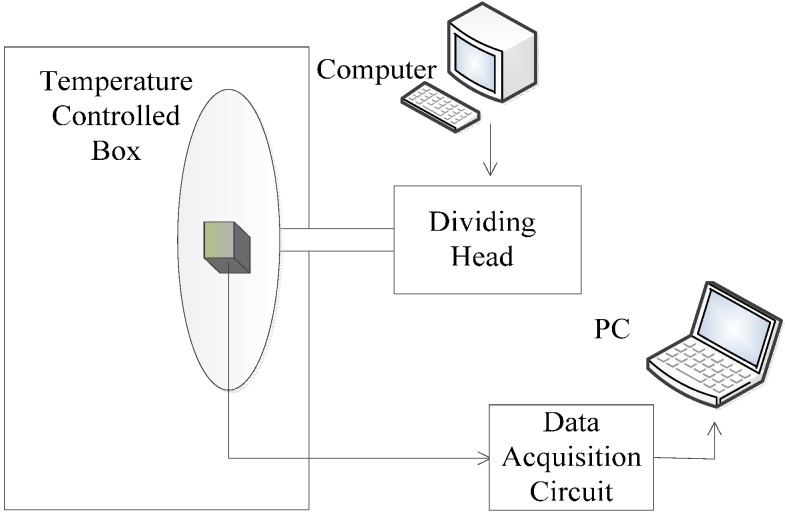
Illustration of the experiment.

**Figure 5:**
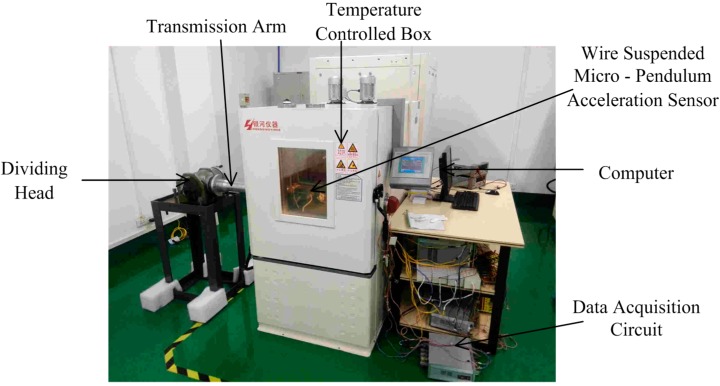
Experimental system.

Figure 4 and Figure 5 demonstrate the schematic diagram and factual photo of the experiment, respectively. The experiment system is composed of a temperature-controlled box, dividing head, power source, and data acquisition circuit. Temperature control box has a range of −60 °C–+90 °C and accuracy of 0.1 °C. Dividing head’s resolution is 0.36″. A computer controls its dial to ensure accurate and stable angles. A transmission arm connecting the dial and the accelerometer ensures the latter is well aligned in the temperature-controlled box. Data acquisition circuit consists of precise sampling resistance, filtering capacitor and a data collector. When the resistance is 500 Ω, the error does not exceed 1‰, and decoupling capacitor is 1.5 *μf*. The collector is an Agilent digital multimeter (34401A) and works at a 2 Hz sampling rate. Table 1 shows the physical parameters of the Micro-Pendulum Accelerometer. The mass of the coil was measured by a Mettler–Toledo balance (ME204) with the accuracy of ten thousandths of a gram, and remnant magnetization 1.035 T was measured after repeatedly magnetizing and before assembling.

**Table 1:** The parameters of the accelerometer.

Symbol	Physical Quantity	Value
*l*_1_	sensitive length of the coil	10.0 mm
*l*_2_	Cantilever length of the coil	8.0 mm
m	mass of the coil	43.2 mg
l	length of the magnet	8.0 mm
W	width of the magnet	4.8 mm
2d	distance between the two magnets	1.0 mm
h	height of magnet	4.0 mm
*B_0_*	remnant magnetization	1.035 T
*g*	local gravity	9.78984 m/s^2^

The process of the experiment started from the low temperature to high temperature. To make sure that the inside and outside of the sensor were well thermally exchanged, the sensor was powered on after heating for 2 h. At each temperature point, current data was collected in the sequence of −g, 0 g and +g acceleration input. When current temperature testing was over, we powered off the sensor, raised the temperature and heated the sensor before the next temperature testing. Data quantities in different situation are listed in Table 2. The reason for collecting more data at 0 g is due to the calculation of the bias and variance in the statistical significance.

**Table 2:** Data quantities in different states.

	−40 °C	24 °C	60 °C
0 g	2061	1071	1084
−1 g	36	62	98
+1 g	71	94	86

## 5. Results and Discussion

**Figure 6:**
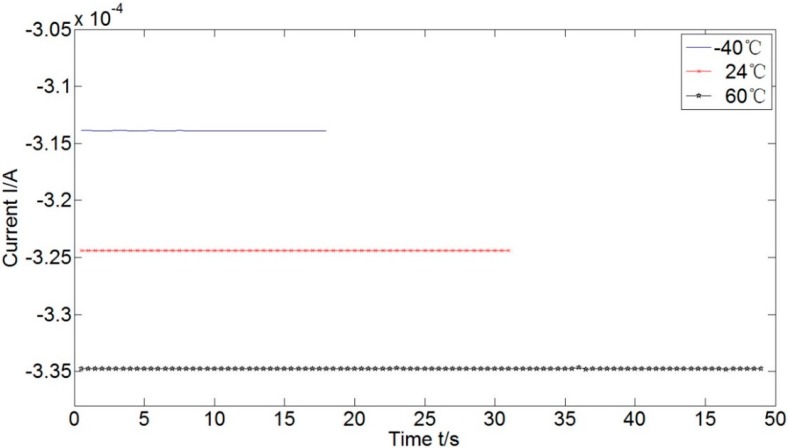
Current outputs of the accelerometer in +g acceleration input.

Figure 6 shows the output of the accelerometer in +g acceleration input. Larger current is acquired at higher working temperatures. The essential reason for this phenomenon is the magnetic field strength decreases when the temperature increases, seeing Equation (5). The coil needs a larger current to generate Ampere moment being equal to inertial moment.

**Figure 7:**
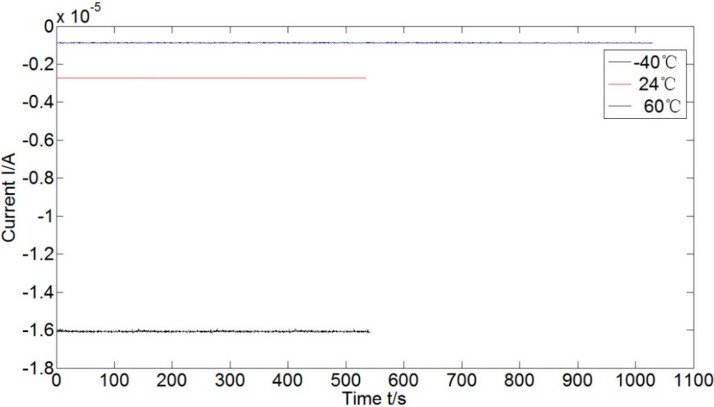
Current bias of the accelerometer at different temperatures.

**Figure 8:**
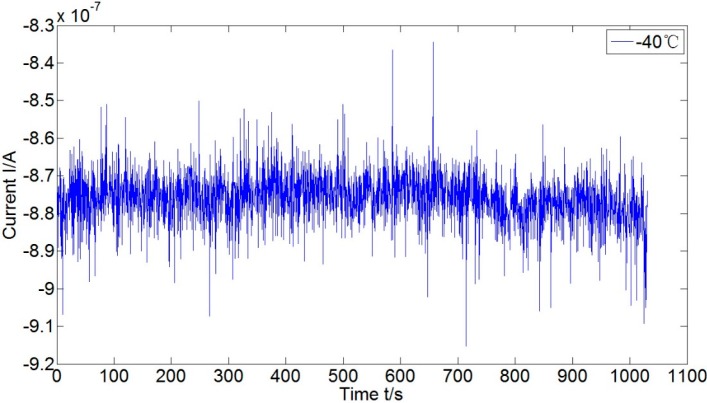
Current bias of the accelerometer at −40 °C.

**Figure 9:**
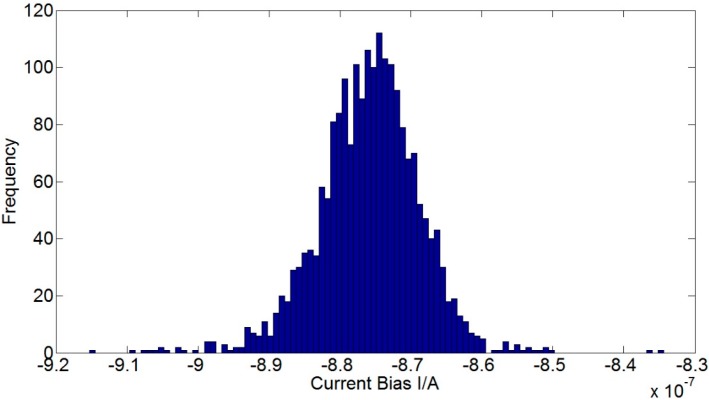
The histogram of current bias.

Adjusting the dividing head to make the accelerometer vertical to gravity direction leads to zero acceleration input of the accelerometer. The corresponding outputs in three testing temperatures are shown in Figure 7. Moreover, the detailed current output in −40 °C and its histogram are shown in Figure 8 and Figure 9, respectively. The current bias increases as the temperature increases, and it is of normal distribution. Calculating the mean and variance of current at different temperatures, we have the results listed in Table 3. The randomness of the current becomes worse in higher temperature. It makes sense if considering in the structural point of view: higher temperature results in weaker filed, and the coil swing is then enhanced as a consequence. The means in Table 3 could be used for the biases compensation at different temperatures, and the variances compose the covariance matrix of noise.

**Table 3:** The mean and variance of the current bias at different temperatures.

Temperature (°C)	Mean (A)	Variance (A^2^)
−40	−8.759 × 10^−7^	2.990 × 10^−17^
24	−2.725 × 10^−6^	5.290 × 10^−17^
60	−1.607 × 10^−5^	8.922 × 10^−16^

Form the matrices in Equation (10) by using the data in different temperature and acceleration combinations, including three temperature points, −40 °C, 24 °C, and 60 °C, and two static accelerations, −g and +g. Three temperature biases and variance elements are given in Table 3. There are 447 epochs in total for the Iterative Least Squares algorithm, according to Table 2. That is, the dimensions of observation *y* and Jacobi matrix *H* are both 447 × 1, and covariance Matrix *R* is 447 × 447. The termination criterion of iteration is that the correction for the estimated remnant magnetization and does not exceed 10^−6^ T. The initial values, iteration numbers, and final estimates are listed in Table 4. Apparently, the initial value of iteration does not affect the estimation accuracy and convergence of the algorithm is good. It is partly because the current bias is estimated before the iteration, so the estimator is reduced to one order. The final estimation of remnant magnetization was 1.035 T. Compared to the true value 1.035 T, the error was less than 0.001 T.

**Table 4:** The results of the Iterative Least Squares at different initial situations.

Initial Value	*B*_0_ (T)	Iteration Number
0.1	1.03494845	9
0.5	1.03494841	6
1.0	1.03494844	3
1.1	1.03494844	4
1.3	1.03494844	5

## 6. Conclusions

For an independently developed Wire Suspended Micro-Pendulum Accelerometer, the internal magnetic field is difficult to measure when making the scale factor calibration. This paper proposed an indirect measuring method for the remnant magnetization based on the inverse process of the sensor’s working mechanism. Due to the nonlinearity inherent in measurement and existing noises, the Iterative Least Squares algorithm was used for approximation and adjustment. Different acceleration and temperature combinations were carefully selected in the testing procedures and the data were combined for iteration. Note that the bias demerged from the iteration by computing the current statistics at zero acceleration input. The results indicated that the designed Iterative Least Squares algorithm had good convergence. The estimated remnant magnetization was 1.035 T and its error was less than 0.001 T. The work proves the feasibility of indirect measurement for the magnetic field in engineering, and also facilitates the scale factor and temperature coefficient calibration.
